# The Scottish school leavers cohort: linkage of education data to routinely collected records for mortality, hospital discharge and offspring birth characteristics

**DOI:** 10.1136/bmjopen-2016-015027

**Published:** 2017-07-10

**Authors:** Catherine H Stewart, Ruth Dundas, Alastair H Leyland

**Affiliations:** MRC/CSO Social and Public Health Sciences Unit, University of Glasgow, Glasgow, UK

**Keywords:** Educational attainment, Young adults, Socioeconomic position, Educational gradients in health, Mortality and morbidity, Linked data

## Abstract

**Purpose:**

The Scottish school leavers cohort provides population-wide prospective follow-up of local authority secondary school leavers in Scotland through linkage of comprehensive education data with hospital and mortality records. It considers educational attainment as a proxy for socioeconomic position in young adulthood and enables the study of associations and causal relationships between educational attainment and health outcomes in young adulthood.

**Participants:**

Education data for 284 621 individuals who left a local authority secondary school during 2006/2007–2010/2011 were linked with birth, death and hospital records, including general/acute and mental health inpatient and day case records. Individuals were followed up from date of school leaving until September 2012. Age range during follow-up was 15 years to 24 years.

**Findings to date:**

Education data included all formal school qualifications attained by date of school leaving; sociodemographic information; indicators of student needs, educational or non-educational support received and special school unit attendance; attendance, absence and exclusions over time and school leaver destination. Area-based measures of school and home deprivation were provided. Health data included dates of admission/discharge from hospital; principal/secondary diagnoses; maternal-related, birth-related and baby-related variables and, where relevant, date and cause of death. This paper presents crude rates for all-cause and cause-specific deaths and general/acute and psychiatric hospital admissions as well as birth outcomes for children of female cohort members.

**Future plans:**

This study is the first in Scotland to link education and health data for the population of local authority secondary school leavers and provides access to a large, representative cohort with the ability to study rare health outcomes. There is the potential to study health outcomes over the life course through linkage with future hospital and death records for cohort members. The cohort may also be expanded by adding data from future school leavers. There is scope for linkage to the Prescribing Information System and the Scottish Primary Care Information Resource.

**Strengths and limitations of this study**

- The Scottish school leavers cohort provides prospective follow-up of school leavers in Scotland for studying associations and causal relationships between educational attainment and health in young adulthood.
- This resource provides access to a large, representative cohort with the ability to study rare health outcomes, such as suicide.
- The cohort is only representative of local authority school leavers and does not include individuals that attended privately funded schools. Although this proportion is low, pupil numbers are not evenly distributed across Scotland, with numbers of private school pupils accounting for a greater proportion of all pupils in some local authority areas than others.
- There may be misestimation of migrants: overestimation for unknown international migrants who are still assumed to be present in the cohort; and underestimation for individuals who have been incorrectly assumed to have migrated to elsewhere in the UK and have been removed from the cohort.

## Introduction

The WHO recognises that reducing health inequalities among countries and among groups within countries is pivotal in achieving the aim of ‘health for all in the 21^st^ century’.^1–3^ To effectively monitor the extent of inequality, they advise that vital statistics from all socioeconomic strata are required.^4^ Socioeconomic factors are known to contribute to inequalities, with those of lower socioeconomic position (SEP) more likely to suffer adverse health outcomes.^5–9^ Commonly used measures of SEP in adulthood include occupational social class^9–11^ and income.^9 10^ However, such measures may not be appropriate for adolescents or young adults.^12–15^ Education is potentially a better measure of SEP in young people. It avoids problems associated with occupational social class or income as education level may be determined for everyone.^16 17^ Education can determine future employment opportunities and income and is more likely to remain stable over a person’s lifetime and be less sensitive to changes in health status during adulthood than other measures of SEP.^18^ Furthermore, education allows for greater international comparability of SEP.^18^

There is a positive association between education and health,^19 20^ with the better educated experiencing more favourable health outcomes such as better self-reported health, lower levels of morbidity and disability, and longer life expectancy.^19 20^ Increased education generally also leads to decreased participation in unhealthy behaviours such as smoking and excess alcohol consumption and increased participation in healthy behaviours such as physical exercise and receiving preventative medical care, all of which can protect health.^19 20^ Education has also been identified as an important factor underlying inequalities in health.^21^

Linkage of education and health data allows for the role of educational attainment on a range of health outcomes in young adulthood to be studied. Problems with representativeness and power for studying rarer outcomes, such as suicide, can be avoided by using population data. Population data provide the most representative view and greatest number of events for common causes of hospitalisation and death in young adulthood and allow for long-term follow-up. Linking education data with hospital and death records for the population of Scottish school leavers allows access to a large representative cohort for investigating education, a proxy for SEP, as a contributory factor to inequalities in health in young adulthood as well as the early years by looking at offspring birth weight. This will help determine whether intervening at school and targeting underachievers would potentially improve the health of young people and their children and thus contribute to a reduction in health inequalities.

This paper describes the data sources used to create the Scottish school leavers cohort and aims to highlight the potential of this linked data resource for investigating socioeconomic differences in the health of young adults. Initial findings for cause-specific morbidity and mortality by educational attainment are presented in the form of crude rates.

## Cohort description

### Inclusion criteria

The cohort was formed by identifying individuals leaving all local authority mainstream secondary and special schools in Scotland during the period 2006/2007 to 2010/2011 from the School Leavers Survey.^22 23^ The School Leavers Survey seeks to obtain accurate data on pupils in each school year who leave school having reached the minimum school leaving age or who have exceptional permission to leave school before the minimum leaving age. Pupils in Scotland can leave school in May if they turn 16 years old between March and September or in December if they turn 16 years old between October and February.^24^ The data are held by the Scottish Exchange of Education Data (ScotXed) unit within the Education Analytical Services Division of the Learning and Justice Directorate of the Scottish Government. Local authorities are responsible for submitting data collected by each school within their area to ScotXed. Information collected includes date of leaving, stage of secondary school at leaving and reason for leaving, as well as a range of demographic information, such as gender and ethnicity. The survey is run biannually in order to cover both winter and summer leaving dates during the academic year.

**Figure 1:**
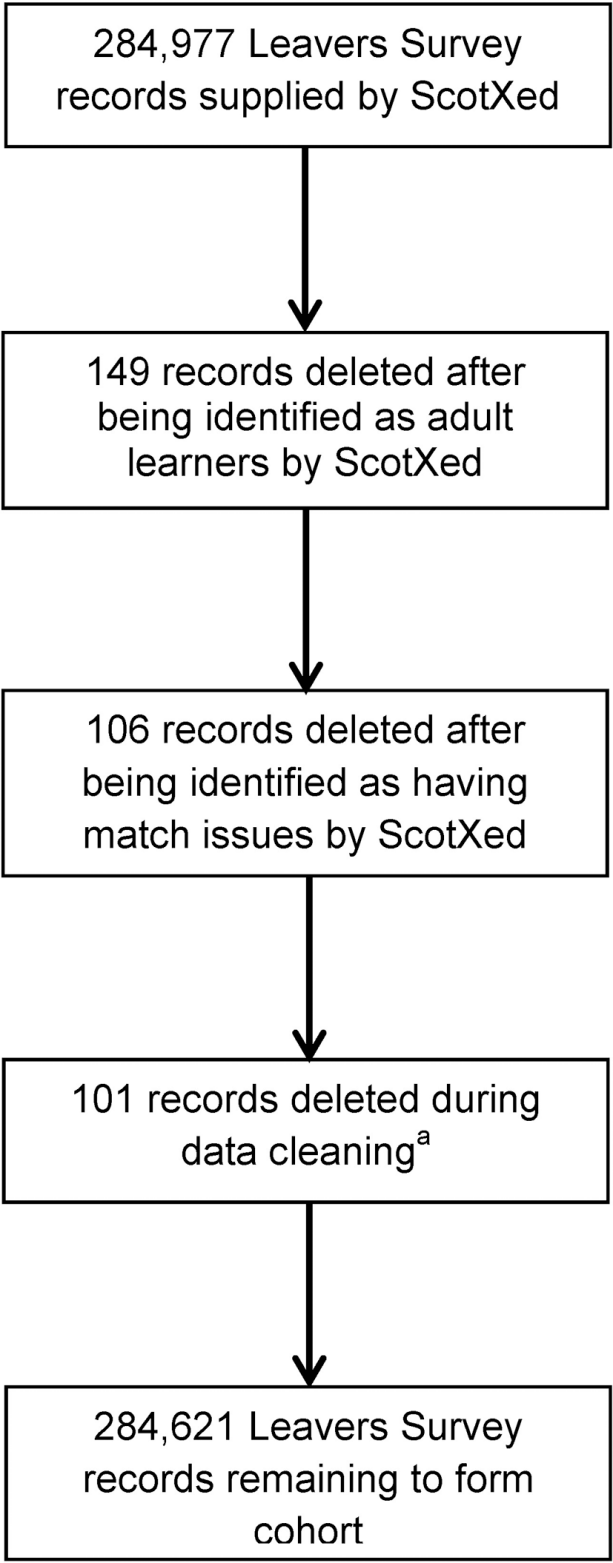
Flow chart of exclusions to form the final cohort. ^a^Reasons for exclusions included: (1) individual was included in the School Leavers Survey, but died prior to the date of leaving recorded in the survey. Possible delays in updating school records; (2) possible false links between education and health records. ScotXed, Scottish Exchange of Education Data.

A total of 284 977 leavers over the 5 year study period were identified from the School Leavers Survey to form the cohort. Of these, 149 and 106 records were excluded after ScotXed later identified them as being adult learners or having match issues with their records, respectively (figure 1). A further 101 records were excluded during the data cleaning process as the individual had died before their official school leaving date as recorded in the survey—a possible result of delays in updating school records, or due to possible false links between education and health records. This left records for 284 621 (99.9% of original total) leavers in the cohort.

The age range of the cohort was approximately 14–20 years reflecting the decision of some pupils to leave on reaching the minimum school leaving age (or have been granted exceptional permission to leave) and others to continue with their studies into upper secondary.

### Education data

Comprehensive educational information, including sociodemographic information, indicators of student needs, educational or non-educational support received and special school unit attendance, attendance, absence and exclusion data over time and school leaver destination information on cohort members was obtained by combining attainment data and data collected from other school surveys. In addition to the School Leavers Survey, these included the School Pupil Census, the National Statistics School Leaver Destinations Survey and the Attendance, Absence and Exclusions Survey. The School Pupil Census is an annual survey used to obtain data on publicly funded schools in Scotland and their pupils. Data are collected from school management information systems for all local authority and grant-aided schools and school centres. These data are generally of high quality since information is recorded directly from pupil enrolment forms.^25^ The National Statistics School Leaver Destinations Survey is used to collect information on the destination of pupils 3 months and then again at approximately 9 months after leaving school. This survey is conducted annually and is used only to obtain information on pupils who attended publicly funded schools in Scotland. The Attendance and Absence and Exclusions Surveys collect data on attendance, absence and exclusions from all local authority schools and mainstream grant aided schools in Scotland. From 2012 data collection for these surveys was changed from annually to every 2 years.^26^

An area-based measure of deprivation for both home and school postcode was also available using the income domain of the 2009 Scottish Index of Multiple Deprivation.^27^

Education data sets were linked using Scottish Candidate Number (SCN). The SCN is the key unique identifier within the Scottish education system. In 2006 all pupils in Scottish primary and secondary schools were assigned an SCN. Each year since, new SCNs have been allocated to primary school pupils on enrolment, to pupils re-entering the Scottish education system having originally started before SCNs were issued and to pupils at any stage entering the Scottish education system for the first time.^28^

**Table 1:** Sociodemographic characteristics of school leavers with Destination Survey record and SMR02 maternity record

	Total	Destination record present n (%)	SMR02 record present n (%)
Gender			
Male†	144 047	133 792 (92.9)	127 892 (88.8)
Female	139 695	130 126 (93.2)*	1 24 117 (88.8)
Ethnicity			
White UK†	263 576	246 183 (93.4)	240 432 (91.2)
Other	13 851	122 243 (88.4)**	6722 (48.5)**
Not known/undisclosed	6315	5492 (87.0)**	4855 (76.9)**
SMR02 maternity record‡			
Present†	252 411	235 730 (93.4)	
Absent	32 210	28 188 (87.5)**	
Attainment (highest SCQF level)§			
No passes at level 3 or better†—no formal school attainment	11 043	7823 (70.8)	8907 (80.7)
SCQF level 3	7972	6930 (86.9)**	7235 (90.8)**
SCQF level 4	52 088	47 549 (91.3)**	47 554 (91.3)**
SCQF level 5	73 001	66 008 (90.4)**	65 663 (89.9)**
SCQF level 6	95 965	91 399 (95.2)**	84 830 (88.4)**
SCQF level 7—high school attainment	44 552	44 209 (99.2)**	38 222 (85.8)**
Residential SIMD at school leaving¶			
Decile 1—most deprived†	32 150	29 235 (90.9)	28 705 (89.3)
Decile 2	29 047	26 680 (91.9)**	26 488 (91.2)**
Decile 3	28 246	26 047 (92.2)**	25 631 (90.7)**
Decile 4	27 544	25 468 (92.5)**	24 865 (90.3)**
Decile 5	27 374	25 485 (93.1)**	24 349 (88.9)
Decile 6	27 276	25 478 (93.4)**	23 691 (86.9)**
Decile 7	27 932	26 109 (93.5)**	24 266 (86.9)**
Decile 8	27 962	26 330 (94.2)**	24 366 (87.1)**
Decile 9	27 825	26 298 (94.5)**	24 665 (88.6)*
Decile 10—least deprived	26 920	25 480 (94.7)**	23 759 (88.3)**
School SIMD¶			
Decile 1—most deprived†	13 247	12 057 (91.0)	11 997 (90.6)
Decile 2	23 503	21 487 (91.4)	21 472 (91.4)*
Decile 3	35 736	33 046 (92.5)**	31 916 (89.3)**
Decile 4	31 689	29 020 (91.6)	28 553 (90.1)
Decile 5	25 322	23 511 (92.8)**	22 244 (87.8)**
Decile 6	30 964	28 873 (93.2)**	26 954 (87.0)**
Decile 7	39 165	36 452 (93.1)**	35 065 (89.5)*
Decile 8	24 347	22 858 (93.9)**	21 137 (86.8)**
Decile 9	29 370	27 148 (92.4)**	26 270 (89.4)**
Decile 10—least deprived	31 278	29 466 (94.2)**	26 803 (85.7)**

Univariate logistic regression was used to investigate the effect of each of the socio-demographic variables in turn onwhether a destination record and maternity record was present or not.

*p<0.05

**p<0.001

†Reference category.

‡Potential indicator of whether individual was born in Scotland (SMR02 maternity record present) or not (SMR02 maternity record absent).

§Scottish Credit and Qualifications Framework (SCQF) level descriptors are presented in table 2.

¶Area deprivation measured using 2009 Scottish Index of Multiple Deprivation (SIMD) income domain deciles.

SMR, maternity inpatient and day case discharges Scottish Morbidity Records.

Of 284 621 leavers, 283 742 (99.7%) were further linked to a School Pupil Census record^29^; 284 621 (100%) to an Attendance, Absence and Exclusion Survey record^26^ and 263 918 (92.7%) to a National Statistics School Leaver Destinations Survey record.^30^ The percentage of school leavers linked to a destination survey was significantly lower for individuals who were not of white UK ethnic origin (p<0.001) or whose ethnicity was unknown (p<0.001) and there was a clear decrease in the percentage linked as attainment decreased (p_trend_ <0.001) and area deprivation of home address at school leaving increased (p_trend_ <0.001) (table 1).

### Linkage to health data

**Figure 2:**
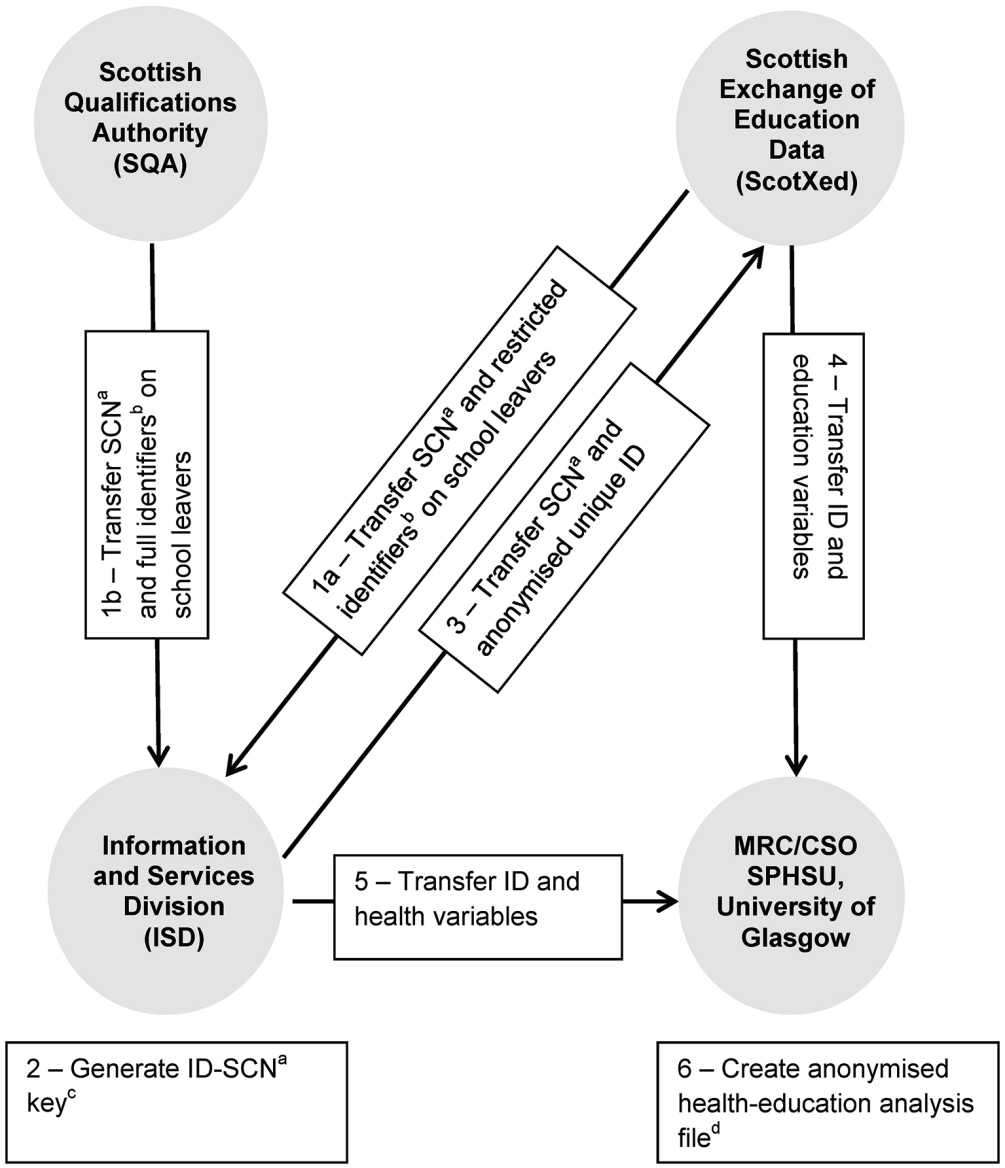
Data flows for linking education and health data ^a^Scottish Candidate Number (SCN). ^b^Restricted identifiers include gender, date of birth and home postcode. Full identifiers include components of restricted identifiers plus forename and surname. ^c^ISD generates an anonymised unique study identifier number for each school leaver. ^d^Analysis file contains only unique ID numbers and no other personal identifiers. MRC/CSO SPHSU, Medical Research Council/Chief Scientist Office Social and Public Health Sciences Unit.

Prospective follow-up of participants’ health outcomes by educational attainment at date of school leaving was provided by linking education data with morbidity and death records. For each of the 284 621 cohort members identified by the School Leavers Survey, data linkage (figure 2) was performed by the Information and Services Division (ISD) of National Health Service (NHS) Scotland using established probability matching techniques based on the Howard Newcombe principles.^31 32^ Personal identifiers, including name, date of birth (DOB), gender and home postcode were supplied by ScotXed and the Scottish Qualifications Authority (SQA) for use in the linkage process. A national database of deaths and Scottish Morbidity Records (SMRs) is maintained by ISD.^33^ SMR data are approximately 90% accurate in the clinical coding of main condition/diagnosis based on the International Classification of Diseases version 10 (ICD-10) codes.^33 34^ SMR schemes linked for the cohort included general/acute inpatient and day case discharges (SMR01); maternity inpatient and day case discharges (SMR02) and mental health inpatient and day case discharges (SMR04). Information on all deaths and hospital episodes extracted from the various SMR schemes, including date of admission and discharge and diagnosis is linked together to provide individual patient records^35^ using the Community Health Index (CHI) number. The CHI is a population register for healthcare purposes in Scotland and assigns a unique identifying number to every person registered with a general practitioner (GP) in Scotland.^36 37^

The cohort includes school leavers who were born outside Scotland. Maternity and hospital records from birth until entry into Scotland for these individuals were not available. Of the 284 621 leavers in the cohort, 252 411 (89%) were linked to a maternity record. Other reasons for missing maternity records include being unable to make a robust link using available identifiers and home births; however, fewer than 1% of births take place outside of hospital in Scotland.^38^ The percentage of school leavers with a missing maternity record was greater among individuals who were not of white UK ethnic origin (p<0.001) or whose ethnicity was unknown (p<0.001) (table 1).

In addition to requesting maternity records (SMR02) for school leavers, such records for any children born to female members of the school leavers’ cohort between the date of school leaving and the end of follow-up were also requested. This allows the transgenerational effects of school education on the health of offspring to be studied.

### Ethical approval and governance permissions

As the cohort was anonymised and formed using secondary data sources, ethical approval was not required. Further, consent of cohort members was not required for data linkage under the Data Protection Act.^39^ However, separate data applications were made to each of the three organisations providing data (SQA, ScotXed and ISD). Data sharing agreements were then issued by SQA and ScotXed for pupil identifier data and education survey data, respectively. As this cohort evolved through linkage of previously unlinked data sets and required access to potentially identifiable data, a further application was made to the Privacy Advisory Committee (PAC) at NHS National Services Scotland (NSS)^40^ in order to access hospital and death records and have them linked with education data. Since applying for these data, procedures for new applications have changed and the NSS PAC no longer receives new applications. New procedures are highlighted in the Collaboration section of this paper.

In order to help protect anonymity of cohort members, the final linked health and education analysis file supplied by ISD was stripped of all personal identifiers and contained unique study identification numbers only.

### Follow-up

School leavers were followed up until September 2012. This date was selected as the end of follow-up at the time of data request in order to ensure the highest level of data completeness (≥95%) for all SMR schemes requested.^41^ Total follow-up time was approximately 964 344 person years with an average follow-up time of approximately 3.4 years. Hospital episodes or deaths occurring between school leaving and September 2012 were the health outcomes to be studied. Health outcome data were unavailable for leavers who moved outwith Scotland. Attempts were made to identify emigrants using the School Leaver Destinations Survey and records indicating deregistration from a GP. A GP deregistration record is generated when individuals register with a new GP elsewhere in the UK. If such a record was present and no further health data were present after this date then it was assumed that the individual had migrated out of Scotland to elsewhere in the UK. Emigration levels in Scotland generally tend to be low.^33^ In this cohort, 499 (0.2%) individuals were identified as emigrants via the School Leaver Destinations Survey. A further 7735 (2.7%) individuals were flagged up as potential emigrants via GP deregistration records.

### Characteristics of school leavers

**Figure 3:**
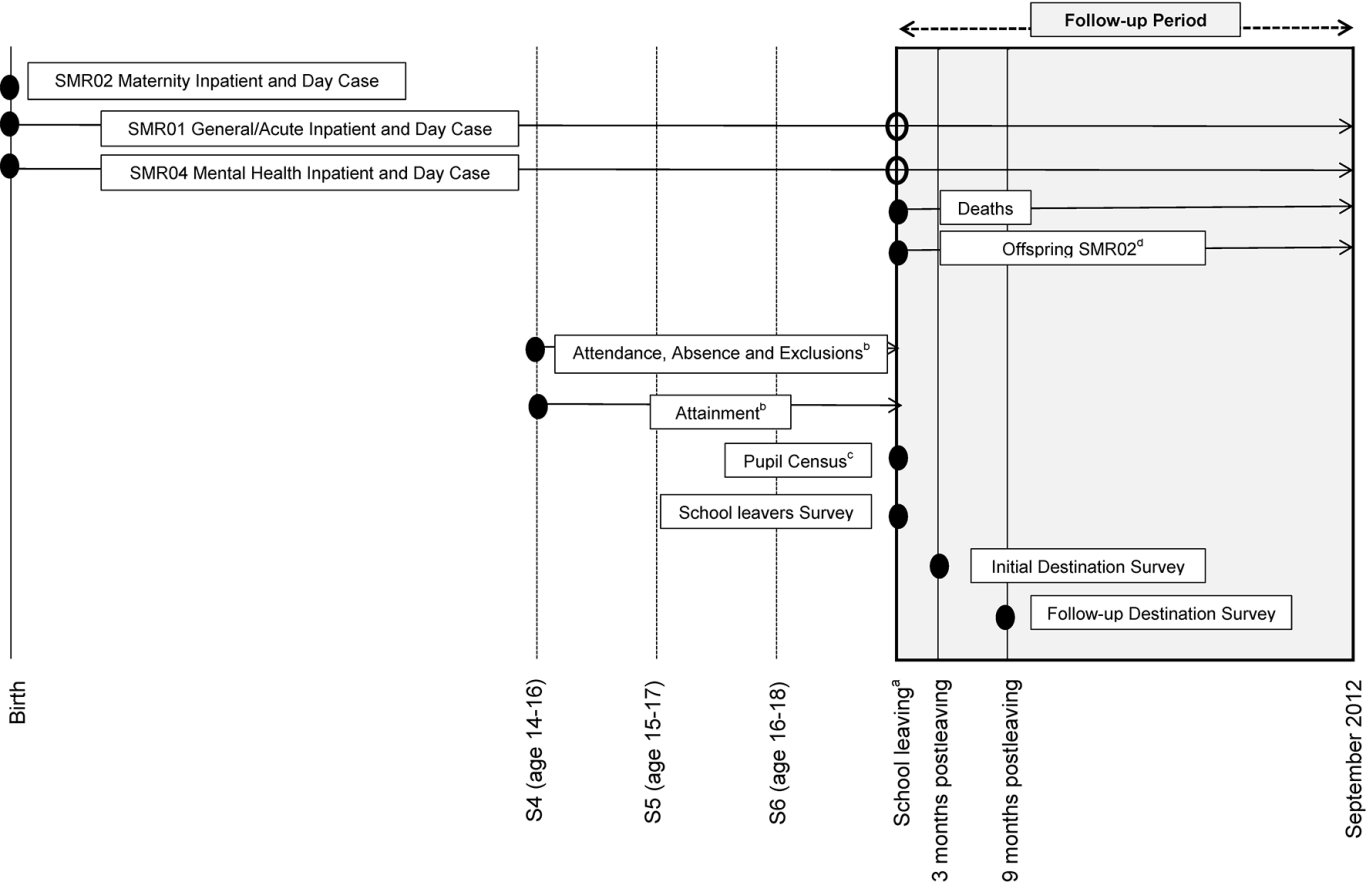
Timeline of health and education data obtained by life stage. ^a^Minimum school leaving age for summer leaving (May) is turning 16 years old between March and September and for winter leaving (December) is turning 16 years old between October and February. Data are for leavers during the period 2006/2007–2010/2011. ^b^Pupils may not have records for all three stages (S4–S6) if they left school prior to end of S6; left school (or took a break from school) at end of S4 and returned again in S6; or transferred to a privately funded school after S4. ^c^Pupil census record corresponds to the most recent record available for an individual prior to school leaving. ^d^SMR02 maternity inpatient and day case records corresponding to all children of female cohort members. SMR, Scottish Morbidity Record.

Figure 3 displays health and education data obtained by life stage of cohort members. Variables extracted from each of the different schemes displayed in figure 3 are presented in box 1.

Box 1 **Variables extracted from education and health data sets for school leavers**

All Scottish Morbidity Record (SMR) and death data schemes*

- Type of record
- Year of birth
- Age (years) at the time of event
- Gender
- Marital status
- Ethnic group
- Deprivation at birth and event: 1981,1991, 2001 Carstairs quintiles; 2006, 2009 and 2012 overall Scottish Index of Multiple Deprivation (SIMD) deciles and income domain SIMD deciles
- Urban/rural indicator

SMR02 maternity inpatient and day case* †

- Date of admission
- Date of discharge
- Maternal age
- Maternal marital status
- Maternal socioeconomic group
- Smoking during pregnancy
- Smoking before pregnancy
- Maternal height
- Outcome of pregnancy
- Number/order of births this pregnancy
- Estimated gestation
- Certainty of gestation
- Apgar Score at 5 min
- Total previous pregnancies
- Outcome of previous pregnancies
- Previous caesarean sections
- Previous neonatal deaths
- Previous spontaneous abortions
- Previous stillbirths
- Previous therapeutic abortions
- Parity
- Baby birth weight
- Baby sex
- Primary and secondary condition/diagnosis

Scottish birth record*

- Birth weight

SMR01 general/acute inpatient and day case and SMR04 mental health inpatient and day case*

- Significant facility code
- Admission type
- Admission reason (SMR01)
- Continuous inpatient stay (SMR01)
- Status on admission (SMR04)
- Previous psychiatric care (SMR04)
- Date of admission
- Date of discharge
- Main condition/principal diagnosis
- Other conditions/diagnosis

**School Leavers Survey‡**

- Reason for leaving
- Date of leaving
- Stage of leaving

**School pupil census‡**

- Gender
- Year of birth
- Ethnic background
- Free school meal registered
- Looked after by local authority (at home or away from home)
- Mainstream integration (in half days)
- Attendance at special schools/units (in half days)
- Nature of additional support provided (educational or non-educational)
- Level of English
- Student need category
- Assessed (by qualified professional) or declared (by parent) disabled

**Attendance, Absence and Exclusions Survey‡**

- Possible attendance by year (in half days)
- Actual attendance by year (in half days)
- Absence by reason and year (in half days)
- Temporary exclusions by reason and year (in half days)
- Ever removed from register

**Attainment‡**

- Grades awarded by subject for Scottish Credit and Qualifications Framework (SCQF) levels 3–7
- Total tariff points§
- Number of courses at SCQF levels 1 and 2

**National Statistics School Leaver Destinations Survey‡**

- Initial destination category
- Follow-up destination category

Death*

- Date of admission
- Date of death
- Cause of death (underlying and contributory)

**Contextual-level variables‡**

- Anonymised school local authority code
- Anonymised school identifier
- Income domain of SIMD deciles for school postcode
- Income domain of SIMD deciles for home postcode at school leaving

*Data obtained from Information and Services Division (ISD) of National Health Service (NHS) Scotland. For detailed lists of all variables and codings available in each of the schemes refer to the ISD Scotland PDF pack available on the Scottish Morbidity Database section of the Administrative Data Liaison Service (ADLS) website http://www.adls.ac.uk/nhs-scotland/scottish-morbidity-database-smr/?detail. It should be noted that this website is no longer updated. For more limited information on SMR schemes refer to SMR crib sheets in the Data Dictionary section of the ISD Scotland website http://www.ndc.scot.nhs.uk/Data-Dictionary/SMR-Crib-Sheets/index.asp.†Variables extracted from SMR02 records corresponding to both cohort members and children of female cohort members.‡Data obtained from ScotXed. For a detailed guide to all information collected in each survey refer to individual survey guides by clicking on links on the ScotXed School Education website http://www.gov.scot/Topics/Statistics/ScotXed/SchoolEducation.
§Tariff points range from 1 to 120 per individual subject depending on course difficulty and award attained. A total score can be calculated by summing all points accumulated during school. Higher scores correspond to higher levels of attainment.

#### Education characteristics of school leavers

Information on all formal school qualifications attained by date of school leaving and other education-related individual-level variables (eg, student needs, attendance) was available. Area deprivation for school postcode and for home postcode at time of birth and school leaving was also available. Attainment data detailing grades awarded by subject according to the Scottish Credit and Qualifications Framework (SCQF) were obtained. There are 12 levels within the framework, where level 12 is the highest. No information on qualifications attained at levels 8 or above was requested as these are generally awarded at college or university. SCQF level 3 is the lowest level at which an external examination is taken. Details of SCQF levels are given in table 2. There has been an overhaul of qualifications since 2013/2014. As this is outside the range of dates for this cohort of leavers (2006/2007–2010/2011), table 2 refers to qualifications taken prior to this change. A comparison of qualifications and how they have changed over time can be found in the SQA SCQF interactive ready reckoner.^42^

**Table 2:** Scottish Credit and Qualifications Framework (SCQF) levels descriptions for attainment data received

Level*	Award	Age, years (stage) usually taken
7	Advanced higher at A–D‡	17–18 (S6)
6	Higher at A–D§	15–18 (S5, S6)
5	Intermediate 2 at A–D¶ **	15–18 (S5, S6)
	Standard grade (credit)** ††	14–16 (S3–S4)
4	Intermediate 1 at A–D¶ ‡‡	15–17 (S5)
	Standard grade (general)†† ‡‡	14–16 (S3–S4)
3†	Standard grade (foundation)†† §§	14–16 (S3–S4)
	Access 3¶ §§	14–16 (S3, S4)
2	Access 2¶ ¶¶ ***	14+ (S3 onwards)
1	Access 1¶ ††† ‡‡‡	14+ (S3 onwards)

*Level 7 corresponds to the most difficult qualification detail requested and level 1 the least difficult.

†Lowest level at which an external examination is taken.

‡New version introduced in 2015/2016.

§New version introduced in 2014/2015.

¶Reached final certification in 2014/2015.

**Replaced with National 5.

††Reached final certification in 2012/2013.

‡‡Replaced with National 4.

§§Replaced with National 3.

¶¶Designed for learners who may have additional learning support needs.

***Replaced with National 2.

†††Designed for learners with severe and profound learning difficulties.

‡‡‡Replaced with National 1.

Descriptive statistics for sociodemographic and educational characteristics of school leavers by highest qualification attained are presented in tables 3, 4, 5 and 6. Table 3 demonstrates that four per cent of school leavers during the period 2006/2007 to 2010/2011 failed to achieve any passes at SCQF level 3 or above. This percentage was significantly higher among boys (4%) than girls (3%) (p<0.001). Although ethnic minority groups significantly outperformed white UK school leavers, with a higher proportion achieving at least one pass at SCQF levels 6 (p<0.001) and 7 (p<0.001) (59% vs 49%), the proportion of leavers with no passes at SCQF level 3 or better was also significantly higher among ethnic minority groups (5% vs 4%, p<0.001).

**Table 3:** Highest Scottish Credit and Qualifications Framework (SCQF) qualification attained at date of leaving school by pupil census characteristics: frequencies (row %)

	No passes at level 3 or better	SCQF level 3	SCQF level 4	SCQF level 5	SCQF level 6	SCQF level 7	Total
Total leavers	11 043 (3.9)	7972 (2.8)	52 088 (18.3)	73 001 (25.7)	95 965 (33.7)	44 552 (15.7)	284 621 (100)
Census record present	10 676 (3.8)	7933 (2.8)	51 975 (18.3)	72 835 (25.7)	95 807 (33.8)	44 516 (15.7)	283 742 (99.7)
Missing census record	367 (41.8)	39 (4.4)	113 (12.9)	166 (18.9)	158 (18.0)	36 (4.1)	879 (0.3)
Sex							
Male†	6261 (4.3)	4834 (3.4)	28 643 (19.9)	40 296 (28.0)	44 001 (30.5)	20 012 (13.9)	144 047 (50.6)
Female	4415 (3.2)**	3099 (2.2)**	23 332 (16.7)**	32 539 (23.3)**	51 806 (37.1)**	24 504 (17.5)**	139 695 (49.1)
Ethnic group							
White UK†	9459 (3.6)	7270 (2.8)	48 891 (18.5)	68 249 (25.9)	88 568 (33.6)	41 139 (15.6)	263 576 (92.6)
Other	710 (5.1)**	349 (2.5)	1672 (12.1)**	2954 (21.3)**	5492 (39.7)**	2674 (19.3)**	13 851 (4.9)
Not known/undisclosed	507 (8.0)**	314 (5.0)**	1412 (22.4)**	1632 (25.8)	1747 (27.7)**	703 (11.1)**	6315 (2.2)
Free school meal registered							
No†	7067 (2.8)	5148 (2.0)	40 725 (16.2)	64 379 (25.6)	91 035 (36.2)	43 438 (17.3)	25 1792 (88.5)
Yes	3609 (11.3)**	2785 (8.7)**	11 250 (35.2)**	8456 (26.5)*	4772 (14.9)**	1078 (3.4)**	31 950 (11.2)
Looked after by local authority							
No†	9036 (3.3)	6930 (2.5)	49 987 (18.0)	72 070 (25.9)	95 587 (34.4)	44 476 (16.0)	278 086 (97.7)
Yes—at home	1182 (34.5)**	712 (20.8)**	1100 (32.1)**	340 (9.9)**	79 (2.3)**	16 (0.5)**	3429 (1.2)
Yes—away from home	458 (20.6)**	291 (13.1)**	888 (39.9)**	425 (19.1)**	141 (6.3)**	24 (1.1)**	2227 (0.8)
Special school unit attendance							
No†	7601 (2.7)	6898 (2.5)	50 353 (18.2)	72 337 (26.1)	95 682 (34.5)	44 467 (16.0)	277 344 (97.4)
0.5—2.5 school days per week	187 (10.8)**	226 (13.1)**	803 (46.5)**	364 (21.1)**	101 (5.8)**	47 (2.7)**	1728 (0.6)
>2.5 school days per week	2888 (61.8)**	809(17.3)**	819 (17.5)	134 (2.9)**	26 (0.6)**	NA** ‡	4678 (1.6)
Student need identified							
No†	7649 (2.8)	5923 (2.2)	47 090 (17.5)	69 845 (26.0)	94 381 (35.1)	44 052 (16.4)	268 940 (94.5)
Yes	3027 (20.5)**	2010 (13.6)**	4885 (33.0)**	2990 (20.2)**	1426 (9.6)**	464 (3.1)**	14 802 (5.2)
Any additional support received							
No†	7634 (2.8)	5920 (2.2)	47 128 (17.5)	69 850 (26.0)	94 251 (35.1)	44 075 (16.4)	268 858 (94.5)
Yes	3042 (20.4)**	2013 (13.5)**	4847 (32.6)**	2985 (20.1)**	1556 (10.5)**	441 (3.0)**	14 884 (5.2)
Assessed or declared disabled							
No†	10405 (3.7)	7849 (2.8)	51 808 (18.3)	72 673 (25.7)	95 686 (33.8)	44 477 (15.7)	282 895 (99.4)
Yes	271 (32.0)**	84 (9.9)**	167 (19.7)	162 (19.1)**	121 (14.3)**	42 (5.0)**	847 (0.3)

Univariate logistic regression was used to investigate whether highest qualification attained differed across levels of each of the characteristics collected by the pupil census.

* p<0.05

**p<0.001

†Reference category.

‡Merged with SCQF 6 as n<10.

**Table 4:** Highest Scottish Credit and Qualifications Framework (SCQF) qualification attained at date of leaving school by Attendance, Absence and Exclusions Survey characteristics: frequencies (row %)

	No passes at level 3 or better	SCQF level 3	SCQF level 4	SCQF level 5	SCQF level 6	SCQF level 7	Total
Total leavers	11 043 (3.9)	7972 (2.8)	52 088 (18.3)	73 001 (25.7)	95 965 (33.7)	44 552 (15.7)	28 4621 (100)
Record present	10 673 (3.8)	7787 (2.8)	51 626 (18.3)	72 475 (25.7)	95 452 (33.8)	44 362 (15.7)	28 2375 (99.2)
Missing record	370 (16.5)	185 (8.2)	462 (20.6)	526 (23.4)	513 (22.8)	190 (8.5)	2246 (0.8)
Ever temporarily or permanently removed from school register							
No†	7405 (3.0)	4337 (1.7)	37 310 (14.9)	63 993 (25.6)	92 674 (37.1)	43 948 (17.6)	24 9673 (87.7)
Yes	3268 (10.0)**	3450 (10.6)**	14 316 (43.8)**	8482 (25.9)	2778 (8.5)**	414 (1.3)**	32 710 (11.5)
Mean attendance and absence rates							
Average attendance rate	65.2**	65.5	77.2	84.5	89.7	93	85
Average authorised absence rate	22.6**	21.8	17.4	12.7	8.8	6	11.8
Average unauthorised absence rate	11.2**	11.3	4.9	2.6	1.6	1	3

Univariate logistic regression was used to investigate whether highest qualification attained differed across levels of the school exclusion indicator variable. Univariate linear regression was used to test for trend in average attendance rates by attainment.

*p/p_trend_<0.05

**p/p_trend_<0.001

†Reference category.

**Table 5:** Highest Scottish Credit and Qualifications Framework (SCQF) qualification attained at date of leaving by home deprivation at birth and school leaving and school deprivation: frequencies (row %)

	No passes at level 3 or better	SCQF level 3	SCQF level 4	SCQF level 5	SCQF level 6	SCQF level 7	Total
Home deprivation at birth†							
Decile 1—most deprived	3237 (8.0)**	2477 (6.1)**	12 199 (30.2)**	11 734 (29.0)**	8765 (21.7)**	2012 (5.0)**	40 424 (14.2)
Decile 2	1645 (5.2)	1454 (4.6)	8262 (26.3)	9380 (29.9)	8284 (26.4)	2356 (7.5)	31 381 (11.0)
Decile 3	1012 (3.7)	919 (3.4)	6199 (22.8)	7938 (29.2)	8383 (30.8)	2769 (10.2)	27 220 (9.6)
Decile 4	737 (2.9)	669 (2.7)	5136 (20.4)	7277 (28.9)	8328 (33.1)	3049 (12.1)	25 196 (8.9)
Decile 5	573 (2.5)	530 (2.3)	4144 (17.8)	6484 (27.8)	8143 (34.9)	3469 (14.9)	23 343 (8.2)
Decile 6	465 (2.1)	397 (1.8)	3195 (14.6)	5573 (25.5)	8322 (38.1)	3867 (17.7)	21 819 (7.7)
Decile 7	373 (1.8)	257 (1.2)	2707 (12.9)	4928 (23.4)	8453 (40.1)	4349 (20.6)	21 067 (7.4)
Decile 8	292 (1.5)	202 (1.0)	2075 (10.5)	4196 (21.3)	8212 (41.6)	4757 (24.1)	19 734 (6.9)
Decile 9	211 (1.1)	134 (0.7)	1634 (8.4)	3735 (19.3)	8326 (43.0)	5329 (27.5)	19 369 (6.8)
Decile 10—least deprived	195 (1.1)	90 (0.5)	1289 (7.0)	3344 (18.1)	7994 (43.3)	5534 (30.0)	18 446 (6.5)
Missing	2303 (6.3)	843 (2.3)	5248 (14.3)	8412 (23.0)	12 755 (34.8)	7061 (19.3)	36 622 (12.9)
Home deprivation at school leaving date†							
Decile 1—most deprived	3132 (9.7)**	2221 (6.9)**	9851 (30.6)**	9109 (28.3)**	6583 (20.5)**	1254 (3.9)**	32 150 (11.3)
Decile 2	1815 (6.3)	1527 (5.3)	8057 (27.7)	8727 (30.0)	7091 (24.4)	1830 (6.3)	29 047 (10.2)
Decile 3	1323 (4.7)	1161 (4.1)	6993 (24.8)	8457 (29.9)	7969 (28.2)	2343 (8.3)	28 246 (9.9)
Decile 4	1041 (3.8)	842 (3.1)	5925 (21.5)	7991 (29.0)	8743 (31.7)	3002 (10.9)	27 544 (9.7)
Decile 5	825 (3.0)	599 (2.2)	5304 (19.4)	7716 (28.2)	9299 (34.0)	3631 (13.3)	27 374 (9.6)
Decile 6	653 (2.4)	529 (1.9)	4316 (15.8)	7176 (26.3)	10 011 (36.7)	4591 (16.8)	27 276 (9.6)
Decile 7	575 (2.1)	375 (1.3)	3823 (13.7)	6803 (24.4)	10 871 (38.9)	5485 (19.6)	27 932 (9.8)
Decile 8	452 (1.6)	299 (1.1)	3107 (11.1)	6277 (22.5)	11 242 (40.2)	6585 (23.6)	27 962 (9.8)
Decile 9	387 (1.4)	182 (0.7)	2410 (8.7)	5508 (19.8)	12 073 (43.4)	7265 (26.1)	27 825 (9.8)
Decile 10—least deprived	335 (1.2)	122 (0.5)	1812 (6.7)	4673 (17.4)	11 543 (42.9)	8435 (31.3)	26 920 (9.5)
Missing	505 (21.5)	115 (4.9)	490 (20.9)	564 (24.1)	540 (23.0)	131 (5.6)	2345 (0.8)
School deprivation†							
Decile 1—most deprived	1204 (9.1)**	789 (6.0)**	3322 (25.1)**	3627 (27.4)**	3380 (25.5)**	925 (7.0)**	13 247 (4.7)
Decile 2	1600 (6.8)	1164 (5.0)	5244 (22.3)	6321 (26.9)	6976 (29.7)	2198 (9.4)	23 503 (8.3)
Decile 3	1662 (4.7)	1309 (3.7)	7632 (21.4)	10 194 (28.5)	11 033 (30.9)	3906 (10.9)	35 736 (12.6)
Decile 4	1253 (4.0)	828 (2.6)	6464 (20.4)	8650 (27.3)	10 356 (32.7)	4138 (13.1)	31 689 (11.1)
Decile 5	799 (3.2)	693 (2.7)	4766 (18.8)	6668 (26.3)	8739 (34.5)	3657 (14.4)	25 322 (8.9)
Decile 6	910 (2.9)	680 (2.2)	5160 (16.7)	7784 (25.1)	11 095 (35.8)	5335 (17.2)	30 964 (10.9)
Decile 7	1122 (2.9)	936 (2.4)	6911 (17.6)	10 117 (25.8)	13 693 (35.0)	6386 (16.3)	39 165 (13.8)
Decile 8	814 (3.3)	437 (1.8)	3906 (16.0)	5930 (24.4)	8671 (35.6)	4589 (18.8)	24 347 (8.6)
Decile 9	1087 (3.7)	729 (2.5)	5018 (17.1)	7182 (24.5)	10147 (34.5)	5207 (17.7)	29 370 (10.3)
Decile 10—least deprived	592 (1.9)	407 (1.3)	3665 (11.7)	6528 (20.9)	11875 (38.0)	8211 (26.3)	31 278 (11.0)

Univariate logistic regression was used to investigate whether highest qualification attained differed across levels of deprivation.

*p_trend_ <0.05

**p_trend_ <0.001

†Based on 2009 Scottish Index of Multiple Deprivation (SIMD) income domain.

**Table 6:** School leaver destination survey characteristics by highest Scottish Credit and Qualifications Framework (SCQF) qualification attained at date of leaving school: frequencies (column %)

	No passes at level 3 or better	SCQF level 3	SCQF level 4	SCQF level 5	SCQF level 6	SCQF level 7	Total
Total leavers	11 043 (3.9)	7972 (2.8)	52 088 (18.3)	73 001 (25.7)	95 965 (33.7)	44 552 (15.7)	284 621 (100)
Leaver destination record present	7823 (70.8)	6930 (86.9)	47 549 (91.3)	66 008 (90.4)	91 399 (95.2)	44 209 (99.2)	263 918 (92.7)
Missing leaver destination record	3220 (29.2)	1042 (13.1)	4539 (8.7)	6993 (9.6)	4566 (4.8)	343 (0.8)	20 703 (7.3)
Initial leaver destinations							
Higher education	44 (0.4)**	16 (0.2)	375 (0.7)	3061 (4.2)	49 035 (51.1)	37 689 (84.6)	90 220 (31.7)
Other education	1808 (16.4)**	1683 (21.1)	16 682 (32.0)	28 004 (38.4)	17 935 (18.7)	2210 (5.0)	68 322 (24.0)
Training & development	1362 (12.3)**	1369 (17.2)	6961 (13.4)	3962 (5.4)	597 (0.6)	59 (0.1)	14 310 (5.0)
Paid employment	950 (8.6)**	880 (11.0)	12 008 (23.1)	22 251 (30.5)	18 040 (18.8)	2868 (6.4)	56 997 (20.0)
Voluntary work	26 (0.2)**	32 (0.4)	112 (0.2)	129 (0.2)	286 (0.3)	215 (0.5)	800 (0.3)
NEET†	3301 (29.9)**	2777 (34.8)	10 708 (20.6)	7872 (10.8)	4891 (5.1)	1008 (2.3)	30 557 (10.7)
Emigrated	45 (0.4)**	20 (0.3)	131 (0.3)	176 (0.2)	136 (0.1)	28 (0.1)	536 (0.2)
Other or unknown	287 (2.6)**	153 (1.9)	572 (1.1)	553 (0.8)	479 (0.5)	132 (0.3)	2176 (0.8)
Follow-up leaver destinations							
Higher education	40 (0.4)**	12 (0.2)	289 (0.6)	2508 (3.4)	45 935 (47.9)	36 639 (82.2)	85 423 (30.0)
Other education	1373 (12.4)**	1286 (16.1)	13 582 (26.1)	24 857 (34.1)	16 742 (17.5)	2107 (4.7)	59 947 (21.1)
Training & development	897 (8.1)**	1013 (12.7)	5057 (9.7)	2723 (3.7)	487 (0.5)	36 (0.1)	10 213 (3.6)
Paid employment	1076 (9.7)**	1012 (12.7)	14 202 (27.3)	25 950 (35.6)	22 632 (23.6)	4029 (9.0)	68 901 (24.2)
Voluntary work	45 (0.4)	44 (0.6)	160 (0.3)	178 (0.2)	315 (0.3)	244 (0.6)	986 (0.4)
NEET†	3878 (35.1)**	3258 (40.9)	13 030 (25.0)	8638 (11.8)	4442 (4.6)	1001 (2.3)	34 247 (12.0)
Emigrated	59 (0.5)**	31 (0.4)	178 (0.3)	218 (0.3)	206 (0.2)	38 (0.1)	730 (0.3)
Other or unknown‡	455 (4.1)**	274 (3.4)	1051 (2.0)	936 (1.3)	640 (0.7)	115 (0.3)	3471 (1.2)

Univariate logistic regression was used to test for trend in leaver destination by attainment.

*p_trend_ <0.05

**p_trend_ <0.001

†Not in education, employment or training.

‡Includes 22 deceased individuals.

NEET, not in education, employment or training.

The number of school leavers failing to achieve any passes at SCQF level 3 or above was also significantly higher for those who received free school meals (11%, p<0.001), were looked after by the local authority either at home (35%, p<0.001) or away from home (21%, p<0.001), attended a special school unit (11% for 0.5–2.5 school days, p<0.001 and 62% for more than half of school days per week, p<0.001), were identified as having additional learning needs (21%, p<0.001), received extra educational or non-educational learning support (20%, p<0.001), were assessed or declared disabled (32%, p<0.001) and had ever been temporarily or permanently excluded from school (10% (table 4), p<0.001).

A significantly increasing trend in educational attainment and average attendance rate (p<0.001) was observed in table 4, with an average attendance rate of 65% for those who failed to achieve any passes at SCQF level 3 or above.

Deprivation at birth was significantly negatively associated with highest SCQF level attained at school leaving (p<0.001; table 5). Eight per cent of school leavers in the most deprived category at birth failed to achieve any passes at SCQF level 3 or above compared with only one per cent in the least deprived category. Deprivation at school leaving had a similar effect on educational attainment (p<0.001). Those who attended schools in the most deprived areas were also significantly more likely to fail to achieve any passes at SCQF level 3 or above (9%) when compared with schools in the least deprived areas (2%) (p<0.001). Table 6 presents destinations of school leavers and demonstrates that the proportion entering higher education significantly increased as highest SCQF qualification attained at date of leaving school increased (p<0.001). Correspondingly, the proportion of school leavers not in education, employment or training significantly decreased as the highest SCQF qualification attained at date of leaving school increased (p<0.001).

#### Health characteristics of school leavers

Key data requested from SMR01 and SMR04 schemes included admission type (routine/urgent/emergency), principal and secondary diagnoses, and date of admission and discharge. Mortality records provided information on date of death and primary and secondary causes of death. For birth record data, variables relating to mother, birth and baby were requested from the SMR02 scheme for both cohort members’ birth and births for any children of female cohort members. Birth weight data were requested from the Scottish Birth Record (SBR) scheme. This allowed birth weight to be identified for each individual born from a multiple pregnancy. SBR data were only requested for cohort members and not for children of female cohort members.

Cause-specific morbidity and mortality rates for the cohort by highest qualification attained are presented in tables 7, 8, 9 and 10. Total mortality rate was generally low with a rate of 47 deaths per 100 000 person years at risk (PYAR; table 7). There were clear significant decreasing gradients (p<0.001) across all-cause and cause-specific mortality rates as the highest qualification attained by school leaving increased. The total admission rate for SMR01 admissions was 10 272 admissions per 100 000 PYAR (table 8).

**Table 7:** Cause of death by highest Scottish Credit and Qualifications Framework (SCQF) qualification at school leaving (rates per 100 000 person years at risk)

	Number of events	No passes at level 3 or better	SCQF level 3	SCQF level 4	SCQF level 5	SCQF level 6	SCQF level 7†	Total
Deaths								
All deaths	453**	161	187	68	36	27	23	47
Accidents and assault	186**	35	84	32	17	11	8	19
Suicide (undetermined and self harm)	120**	40	48	20	11	6		12
Other‡	147**	87	55	17	8	10	10	15

Univariate Poisson regression was used to test for trend in each of the health outcomes by attainment.

*p_trend_ <0.05

**p_trend_ <0.001

†Merged with SCQF 6 as n<10

‡Includes deaths with ICD-10 codes other than for accident, assault or suicide.

ICD-10 codes for outcomes presented in the table are as follows: accidents V01–X59, Y85, Y86 and assault X85–Y09; suicide (undetermined and self-harm) X60–X84, Y10–Y34, Y870 Y872.

**Table 8:** General acute inpatient and day case admissions by highest Scottish Credit and Qualifications Framework (SCQF) qualification at school leaving (rates per 100 000 person years at risk)

	Number of events	No passes at level 3 or better	SCQF level 3	SCQF level 4	SCQF level 5	SCQF level 6	SCQF level 7	Total
All admissions postschool leaving	99 027**	16 319	14 739	12 984	11 126	8628	6100	10 272
Intentional self-harm†	4697**	1408	1481	891	476	212	115	487
Assault†	2709**	793	812	533	287	108	63	281
Alcohol-related†	4769**	1361	1697	909	450	210	164	495
Drug misuse†	831**	369	399	175	68	17	11	86
Neoplasms	4038**	868	267	321	366	510	341	419
Malignant	2780**	759	194	188	220	379	226	288
Benign	1164	87	62	118	134	126	111	121
Accidents	9758**	1663	1990	1447	1126	696	544	1012
Road traffic accidents	1740**	248	256	254	227	126	84	180
All other accidents	8018**	1415	1734	1192	899	570	460	831
Diseases of the respiratory system	7773**	892	772	951	926	741	519	806
Acute tonsillitis	3136	176	263	379	379	320	220	325
Other diseases of upper respiratory tract	2271	176	194	244	266	236	193	235
Asthma	910**	112	117	145	129	63	24	94
Diseases of the digestive system	12 110**	1817	1394	1341	1300	1207	980	1256
Diseases of teeth and supporting structures	2619**	605	249	253	258	273	226	272
Diseases of appendix	1477*	154	143	177	171	131	140	153
Crohn's disease and ulcerative colitis	2044**	97	227	178	221	249	190	212
Diseases of the genitourinary system	7393**	793	1101	898	826	706	544	767
Symptoms, signs and abnormal clinical and lab findings, not elsewhere classified	13 286**	2095	2001	1923	1525	1069	733	1378
Abdominal and pelvic pain	7478**	952	1174	1160	874	585	373	775
Pain in throat and chest	1014**	186	157	144	123	82	40	105
Headache	927**	94	117	123	109	83	62	96

Univariate Poisson regression was used to test for trend in each of the health outcomes by attainment.

*p_trend_ <0.05

**p_trend_ <0.001

†Apart from intentional self-harm, assault, alcohol-related, drug misuse and accidents, admission outcomes in the table were defined if the corresponding ICD-10 codes was the primary diagnosis. Admissions for intentional self-harm, assault, alcohol-related, drug misuse and accidents were defined as such if the corresponding ICD-10 codes were primary or secondary diagnoses, therefore double-counting of some events may occur if the primary diagnosis was for something else.

ICD-10 codes for outcomes presented in the table are as follows: intentional self-harm X60–X84, Y870, Y10–Y34, Y872; assault X85–Y09, Y87; alcohol-related E244, E512, F10, G312, G621, G721, I426, K292, K70, K852, K860, O354, P043, Q860, R780, T510, T511, T519, X45, X65, Y15, Y573, Y90, Y91, Z502, Z714, Z721; drug misuse F11, F12, F13, F14, F15, F16, F18, F19; neoplasms C00–C97, D00–D09, D10–D36, D37–D4; malignant neoplasms C00–C97; benign neoplasms D10–D36; accidents V01–X59, Y85, Y86; road traffic accidents V01–V04, V06–V80, V87, V89, V99; diseases of the respiratory system J00–J99; acute tonsillitis J03; other diseases of the upper respiratory tract J30–J39; asthma J45; diseases of the digestive system K00–K93; diseases of teeth and supporting structures K00–K08; diseases of appendix K35–K38; Crohn's disease and ulcerative colitis K50–K51; diseases of the genitourinary system N00–N99; symptoms, signs and abnormal clinical and lab findings, not elsewhere classified R00–R99; abdominal and pelvic pain R100–R104; pain in throat and chest R070–R074; headache R51.

**Table 9:** Mental health inpatient and day case admissions by highest Scottish Credit and Qualifications Framework (SCQF) qualification at school leaving (rates per 100 000 person years at risk)

	Number of events	No passes at level 3 or better	SCQF level 3	SCQF level 4	SCQF level 5	SCQF level 6	SCQF level 7	Total
All admissions postschool leaving	1963**	1020	589	313	156	93	80	204
Alcohol and/or drug-related	300**	117	146	66	23	7	NA†	31
Schizophrenia, schizotypal and delusional disorders	321**	102	95	52	27	19	20	33
Mood (affective) disorders	531**	146	135	90	53	30	24	55
Depressive episode	368**	107	99	63	37	19	18	38
Neurotic, stress-related and somatoform disorders	222**	97	66	39	15	13	9	23
Reaction to severe stress and adjustment disorders	168**	74	55	35	11	6	NA†	17
Disorders of adult personality and behaviour	234**	114	51	43	15	13	9	24
Mental retardation	136**	283	40	6	0	0	0	14
General psychiatric examination and observation for suspected mental and behavioural disorders	242**	131	69	43	23	6	9	25

Univariate Poisson regression was used to test for trend in each of the health outcomes by attainment.

*p_trend_ <0.05

**p_trend_ <0.001

†Merged with SCQF 6 as n<10

ICD-10 codes for outcomes presented in the table are as follows: Alcohol E244, E512, F10, G312, G621, G721, I426, K292, K70, K852, K860, O354, P043, Q860, R780, T510, T511, T519, X45, X65, Y15, Y573, Y90, Y91, Z502, Z714, Z721 and/or drug-related F11, F12, F13, F14, F15, F16, F18, F19; schizophrenia, schizotypal and delusional disorders F20–F30; mood (affective) disorders F30–F39; depressive episode F32–F33; neurotic, stress-related and somatoform disorders F40–F48; reaction to severe stress and adjustment disorders F43–F44; disorders of adult personality and behaviour F60–F69; mental retardation F70–F79; general psychiatric examination Z004 and observation for suspected mental and behavioural disorders Z032.

NA, not applicable.

**Table 10:** Maternity and neonatal outcomes by highest Scottish Credit and Qualifications Framework (SCQF) qualification at school leaving†

	Number of events	No passes at level 3 or better	SCQF level 3	SCQF level 4	SCQF level 5	SCQF level 6	SCQF Level 7	Total
Total pregnancy rate postschool leaving/1000 women‡§	28 357**	440	465	406	266	112	42	203
Incident pregnancy rate post school leaving/1000 women‡§¶	21 493**	300	334	307	211	95	37	158
Live birth rate postschool leaving/1000 women	15 821**	320	342	260	146	44	9	113
Medical abortion rate/1000 women§	11 559**	101	102	131	111	65	31	83
Stillbirth rate post school leaving/1000 total births	85	6††	NA††	6	4	5	0	5
Perinatal death rate postschool leaving/1000 total births‡‡	109	8	NA§§	8	6	5	0	7
Neonatal death rate postschool leaving/1000 live births¶¶	30***	NA†††	NA†††	NA†††	NA†††	NA†††	NA†††	2
Multiple birth rate postschool leaving/1000 maternities‡‡‡	103	6††	NA††	7	7	6	NA§§§	7
Low birth weight incidence rate/100 live births	1081**	8	11	7	6	5	5	7
Mean age at first pregnancy (with abortive or birth outcome)¶	NA**	18	18	18	18	19	19	18

Univariate Poisson regression was used to test for trend in each of the health outcomes by attainment.

*p_trend_ <0.05

**p_trend_ <0.001

†Incident pregnancy rate is defined as (# of women whose first pregnancy was after school leaving)/(total # of women with no history of pregnancy prior to school leaving). Unless stated, all other rates are based on all female cohort members and an individual may contribute more than one event.

‡Total and incident pregnancy rates include live births, stillbirths and spontaneous or medical abortion.

§Information on medical abortion comes from general acute inpatient and day case admissions (SMR01) scheme.

¶Individuals with any known history of pregnancy prior to school leaving have been excluded.

††Categories for no passes at SCQF 3 or better and SCQF 3 have been merged as n<10 in both. Results are presented under no passes at SCQF or better.

‡‡Perinatal deaths refer to stillbirths and early neonatal (live births dying within the first 6 days) deaths.

§§Merged with no passes at SCQF 3 or better as n<10

¶¶Neonatal deaths refer to early neonatal (live births dying within the first 6 days) and late neonatal (live births dying on or after the 7th completed day, but before the 28th day) deaths.

*** A test for trend across SCQF levels has not been performed due to small numbers.

†††Numbers of events were too small to present rates by SCQF level. There were 30 neonatal deaths giving a rate of 2 per 1000 live births.

‡‡‡Maternities refers to live births and stillbirths.

§§§Merged with no passes at SCQF 6 or better as n<10.

NA, not applicable; SMR, Scottish Morbidity Record.

Cause-specific rates were greatest for diseases of the digestive system and accidents (1256 and 1012 admissions per 100 000 PYAR, respectively). There was a significant decreasing trend in rate of admission as highest qualification attained increased for all diagnoses other than benign neoplasms (p=0.070), acute tonsillitis (p=0.380), other diseases of the upper respiratory tract (p=0.881) as well as Crohn’s disease and ulcerative colitis, where rates were significantly increasing as the highest qualification attained increased.

The total admission rate for psychiatric and learning disabilities hospitals and units (SMR04) was 204 admissions per 100 000 PYAR (table 9). The greatest cause-specific admission rate was for mood (affective) disorders (55 admission per 100 000 PYAR), in particular for depressive episodes (38 admissions per 100 000 PYAR). Again, the significant decreasing trend (p<0.001) in admission rates with increasing attainment can be observed for all cause-specific admissions. There were slightly elevated rates in the highest attainment group (SCQF 7) for schizophrenia and general psychiatric examination.

The total pregnancy rate among female cohort members (including live births and stillbirths, deaths and all abortions) was 203 per 1000 women (table 10), of which 158 per 1000 women were first ever pregnancies. Mean age at first pregnancy across the whole cohort was 18 years with the highest qualified women (SCQF 7) experiencing first pregnancy significantly later (age 19 years) than the least qualified (age 18 years) on average. The medical abortion rate was 83 abortions per 1000 women. Total rates of 5 per 1000 total births were observed for stillbirths, 7 per 1000 total births for perinatal deaths and 2 per 1000 live births for neonatal deaths. There was no significant effect of educational attainment on the multiple birth rate, with the overall rate being 7 per 1000 maternities. Finally, there was a significant decreasing trend in low birth weight (<2500 g) rate of offspring as attainment increased, with a rate of 8 low birthweight babies per 100 live births in the least qualified group compared with 5 in the most qualified group (SCQF 7).

## Findings to date and future plans

The Scottish school leavers cohort has involved recent novel linkage of education and health data, therefore key findings and publications to date are limited. Due to the young age of the cohort, research has focused mainly on mental health outcomes. Initial work to date includes investigation of educational attainment as a mediator of the relationship between low birth weight and attempted and completed suicide in young adulthood. This work showed that educational attainment accounted for more than half of the effect of birth weight on attempted and completed suicide in young adults.^43^ Educational attainment remained strongly associated with suicidal behaviour, even on full adjustment for a wide range of early life and maternal, educational and childhood mental and physical health risk factors.

Other work has included investigating educational inequalities in birth weight of first-born children of female cohort members.^44^ This work showed that adjustment for maternal birth weight, childhood health, and educational and antenatal risk factors explained more than half of the effect of educational attainment on the odds of having a low birthweight baby. In particular, this work suggested that targeting young women during pregnancy and improving antenatal risk factors (eg, smoking) has the potential to contribute to a decrease in educational differences in the risk of low birthweight offspring.

Future plans mainly include continuing to study the various roles played by educational attainment on mental health outcomes in young adulthood, such as whether deterioration in attainment over time can predict later adverse mental health outcomes including, for example, suicidal behaviour or substance abuse. The availability of hospital records from birth allows any deterioration in childhood mental and physical health, which may confound associations between the exposure and outcome, to be controlled for. Other future plans may also involve linkage to prescription data and the Scottish Primary Care Information Resource (SPIRE).^45^ These data schemes may provide a more representative view of some health outcomes in young adults, particularly for mental health outcomes given the shift away from inpatient psychiatric care towards primary care centres and community-based settings.^46–48^ SPIRE is a new resource in Scotland for extracting data from GP practice systems.^45^ Expected completion date for Scotland-wide installation of data extraction infrastructure for SPIRE is February 2017.

## Strengths and limitations

### Strengths

The main strength is access to a large, representative, prospective cohort study of all local authority secondary school leavers in Scotland through linkage of comprehensive education data with hospital and death records. Availability of data across the life course allows associations between education and health in young adults, while accounting for a wide range of other sociodemographic, biological, psychological and clinical risk factors to be studied, and causal relationships to be explored. There is the potential to extend the follow-up period through record linkage with future hospital records to study a wider range of health outcomes over the life course of this cohort. This can determine whether the association between education and health changes over the life course. As well as extending the follow-up period, there is also the possibility of extending the cohort by adding data from future leavers.

As attainment data were available by individual subject, there was great flexibility for creating various measures of attainment. In particular, a continuous measure of attainment may be created by calculating tariff scores.^49^ Further, there is also scope for creating measures more suited to making international comparisons of educational attainment. Information on attainment over different school stages allows for comparisons of attainment over time within an individual. Another strength was the ability to access names of pupils to use for data matching when linking with health records. Previous attempts to link education and health data have relied on limited identifiers, using residential postcode, DOB and gender only.^50–53^ Having a name available for this cohort guarantees a more robust match and helps eliminate problematic matching of individuals with the same postcode-DOB-gender identifiers including same-sex multiple births.^50^

### Limitations

No information on educational qualifications taken after school leaving was available, so this study was unable to determine final achieved education. However, school attainment has been shown to be associated with a young person’s likelihood of entering higher education.^54^

Currently, data are not available for school leavers leaving privately funded schools in Scotland. Although only a small proportion (~4%)^55^ of all Scottish school pupils attend privately funded schools, pupil numbers are not evenly distributed across Scotland, with numbers of private school pupils accounting for a greater proportion of all school pupils in some local authority council areas than others.^56^ There are data quality issues regarding the Attendance and Absence Survey. Variations in recording of certain absence reasons, particularly for sickness and truancy, over time and between local authorities means care should be taken when making year to year or local and national-level comparisons.^57^ However, overall attendance and absence rates are not affected.^57^

Information on early life factors is missing for young people born out of Scotland as this information is extracted from birth and maternity records. Childhood mental and physical health problems may be underestimated for such individuals since acute and psychiatric hospital records, which are used to create measures of childhood health, are only available after entry to Scotland. There may also be misestimation of PYAR for rate calculations: overestimation for unknown international emigrants who are still assumed to be present in the cohort; and underestimation for individuals who have been incorrectly assumed to have migrated to elsewhere in the UK and have been removed from the cohort. Lastly, medical abortions carried out in private clinics were not included in rate calculations. However, the risk of underestimation is small as less than 1% of medical abortions in Scotland are performed outwith NHS premises.^58^

## Collaboration

This study has demonstrated that education and health data for school leavers in Scotland can be linked to provide access to a large, representative cohort for studying educational variations in health outcomes in young adulthood. Researchers can apply for access to education and health data by making an application to the data controllers. School education data are held by the Education Analytical Services Division of the Scottish Government, http://www.gov.scot/Topics/Statistics/Browse/School-Education/DataAccess and any queries should be directed to ASUSchools.Data.Access@gov.scot. Health data are held by National Services Scotland http://www.isdscotland.org/Products-and-Services/EDRIS/, queries can be made by email to nss.edris@nhs.net. Researchers will need to seek the relevant permissions to link the data.

## References

[1] Mackenbach JP, Bakker MJ Tackling socioeconomic inequalities in health: analysis of European experiences. Lancet 2003;362:1409–14. 10.1016/S0140-6736(03)14639-9 14585645

[2] World Health Organization. Targets for health for all. Copenhagen: WHO Regional Office for Europe, 1986.

[3] World Health Organization. Health 21: an introduction to the health for all policy framework for the WHO European Region. Copenhagen: WHO Regional Office for Europe, 1998.

[4] Prinja S, Kumar R Reducing health inequities in a generation: a dream or reality? Bull World Health Organ 2009;87:84 10.2471/BLT.08.062695 19274354 PMC2636202

[5] Adler NE, Boyce WT, Chesney MA, et al Socioeconomic inequalities in health: no easy solution. JAMA 1993;269:3140–5. 8505817

[6] Adler NE, Newman K Socioeconomic disparities in health: pathways and policies. Health Aff 2002;21:60–76. 10.1377/hlthaff.21.2.60 11900187

[7] Daly MC, Duncan GJ, McDonough P, et al Optimal Indicators of Socioeconomic Status for Health Research. Am J Public Health 2002;92:1151–7. 12084700 10.2105/ajph.92.7.1151 PMC1447206

[8] Cutler DM, Lleras-Muney A, Vogl T Socioeconomic status and health: dimensions and mechanisms. Cambridge, MA: National Bureau of Economic Research, 2008 Working Paper Series No. 14333.

[9] Galobardes B, Lynch J, Smith GD Measuring socioeconomic position in health research. Br Med Bull 2007;81-82:21–37. 10.1093/bmb/ldm001 17284541

[10] Currie CE, Elton RA, Todd J, et al Indicators of socioeconomic status for adolescents: the WHO health Behaviour in School-aged Children survey. Health Educ Res 1997;12:385–97. 10.1093/her/12.3.385 10174221

[11] Davey Smith G, Hart C, Hole D, et al Education and occupational social class: which is the more important indicator of mortality risk? J Epidemiol Community Health 1998;52:153–60. 10.1136/jech.52.3.153 9616419 PMC1756692

[12] West P Inequalities? Social class differentials in health in British youth. Soc Sci Med 1988;27:291–6. 10.1016/0277-9536(88)90262-6 3175713

[13] West P, Sweeting H Health inequalities: what's going on in youth? Health Educ 1996;5:14–20.

[14] Hanson MD, Chen E Socioeconomic Status and Health Behaviors in Adolescence: a review of the literature. J Behav Med 2007;30:263–85. 10.1007/s10865-007-9098-3 17514418

[15] Galobardes B, Shaw M, Lawlor DA, et al Indicators of socioeconomic position (part 1). J Epidemiol Community Health 2006;60:7–12. 10.1136/jech.2004.023531 PMC2465546 16361448

[16] Christenson BA, Johnson NE Educational inequality in adult mortality: an assessment with death certificate data from Michigan. Demography 1995;32:215–29. 10.2307/2061741 7664961

[17] Elo IT, Preston SH Educational differentials in mortality: United States, 1979-85. Soc Sci Med 1996;42:47–57. 10.1016/0277-9536(95)00062-3 8745107

[18] von dem Knesebeck O, Verde PE, Dragano N Education and health in 22 European countries. Soc Sci Med 2006;63:1344–51. 10.1016/j.socscimed.2006.03.043 16698158

[19] Ross CE, Wu C-ling The links between education and health. Am Sociol Rev 1995;60:719–45. 10.2307/2096319

[20] Brännlund A, Hammarström A, Strandh M, Education SM Education and health-behaviour among men and women in Sweden: a 27-year prospective cohort study. Scand J Public Health 2013;41:284–92. 10.1177/1403494813475531 23404182

[21] Scottish Government. Better health, better care: action plan. Edinburgh: The Scottish Government, 2007.

[22] Scottish Exchange of Education Data (ScotXed) Unit. School leavers (Christmas) Survey background. http://www.gov.scot/Topics/Statistics/ScotXed/SchoolEducation/SchoolLeaversChristmas (accessed 15 Sep 2016).

[23] Scottish Exchange of Education Data (ScotXed) Unit. School leavers (Summer) Survey background. http://www.gov.scot/Topics/Statistics/ScotXed/SchoolEducation/SchoolLeaversSummer (accessed 15 Sep 2016).

[24] Scottish Government. What can I do at my age. 2009 http://www.scotland.gov.uk/Publications/2009/04/02155040/12014).

[25] Administrative Data Liaison Service. Dataset summary: pupils in Scotland census. 2014 http://www.adls.ac.uk/the-scottish-government/pupils-in-scotland-census/?detail2014).

[26] Scottish Exchange of Education Data (ScotXed) Unit. Attendance, absence and exclusions survey background. http://www.gov.scot/Topics/Statistics/ScotXed/SchoolEducation/AttendanceAbsenceExclusions (accessed 15 Sep 2016).

[27] Scottish Government. Scottish index of multiple deprivation 2012: a national statistics publication for Scotland. 2012 http://22fa0f74501b902c9f11-8b3fbddfa1e1fab453a8e75cb14f3396.r26.cf3.rackcdn.com/simd_448749_v7_20121217.pdf (accessed 22 Jan 2016).

[28] Scottish Exchange of Education Data (ScotXed) Unit. The Scottish Candidate Number.

[29] Scottish Exchange of Education Data (ScotXed) Unit. School/Pupil census survey background. http://www.gov.scot/Topics/Statistics/ScotXed/SchoolEducation/SchoolPupilCensus (accessed 15 Sep 2016).

[30] Skills Development Scotland. School Leaver destinations statistics. https://www.skillsdevelopmentscotland.co.uk/publications-statistics/statistics/school-leaver-destinations/?page=1&statisticCategoryId=8&order=date-desc (accessed 15 Sep 2016).

[31] Information Services Division Scotland. eDRIS frequently asked questions: can you explain the data linkage process? http://www.isdscotland.org/Products-and-Services/EDRIS/FAQ-eDRIS/index.asp?Co=Y#e1 (accessed 30 Jan 2017).

[32] Fleming M, Kirby B, Penny KI Record linkage in Scotland and its applications to health research. J Clin Nurs 2012;21(19-20):2711–21. 10.1111/j.1365-2702.2011.04021.x 22985317

[33] Gray L, Batty GD, Craig P, et al Cohort profile: the scottish health surveys cohort: linkage of study participants to routinely collected records for mortality, hospital discharge, Cancer and offspring birth characteristics in three nationwide studies. Int J Epidemiol 2010;39:345–50. 10.1093/ije/dyp155 19349480 PMC2846439

[34] Information Services Division Scotland. Data quality assurance: assessment of SMR01 data Scotland 2014-2015. Scotland: Information Services Division Scotland, 2015.

[35] Craig P The Scottish Health Survey-Scottish Morbidity Record Linked Datasets: introduction to a new resource. www.ccsr.ac.uk/esds/events/2008-07-08/petercraig.ppt2014

[36] Information Services Division Scotland. ISD Scotland data dictionary: chi number. http://www.ndc.scot.nhs.uk/Dictionary-A-Z/Definitions/index.asp?Search=C&ID=128&Title=CHI_Number11 (accessed 11 Jan 2017).

[37] Scottish Informatics Programme. Background: Scotland's unique resources. http://www.scot-ship.ac.uk/overview.html (accessed 11 Jan 2017).

[38] Gray R, Bonellie SR, Chalmers J, et al Social inequalities in preterm birth in Scotland 1980-2003: findings from an area-based measure of deprivation. BJOG 2008;115:82–90. 10.1111/j.1471-0528.2007.01582.x 18053104

[39] Scottish Government. Guiding principles for data linkage. Scotland: Scottish Government, 2012.

[40] NHS National Services Scotland. Privacy advisory committee. https://nhsnss.org/how-nss-works/policies-and-statements/privacy-advisory-committee/ (accessed 20 Jan 2017).

[41] Information and Sevices Division Scotland. SMR completeness from 2009. Scotland: Information and Sevices Division Scotland, 2010.

[42] Scottish Qualifications Authority. SCQF interactive ready reckoner. 2016 http://www.sqa.org.uk/files_ccc/readyreckoner.html (accessed 6 Sep 2016).

[43] Stewart C, Leyland AH Education as a mediator of the relationship between low birthweight and attempted and completed suicide in young adults in Scotland, 2007-2012. Eur J Public Health 2014;24(suppl_2):cku163–044. 10.1093/eurpub/cku163.044

[44] Stewart C, Leyland AH Educational inequalities in offspring birthweight: cohort study of young mothers in Scotland, 2007–12. Eur J Public Health 2015;25(suppl_3):ckv170.053 10.1093/eurpub/ckv170.053

[45] NHS Scotland. Spire: scottish primary care information resource. 2016 http://www.spire.scot.nhs.uk/ (accessed 12 Sep 2016).

[46] Stewart CH Multilevel modelling of event history data: comparing methods appropriate for large datasets. Scotland: University of Glasgow and MRC/CSO Social & Public Health Sciences Unit, 2010.

[47] World Health Organisation. Integrating mental health into primary care: a global perspective. Switzerland: World Health Organisation, 2008.

[48] Scottish Government. Mental health strategy for Scotland 2012-2015. Scotland: Scottish Government, 2012.

[49] Scottish Government. SQA attainment and school Leaver qualifications in Scotland: 2008-09 - Annex A: unified points score scale. 2010 http://www.gov.scot/Publications/2010/03/22111037/4 (accessed 2 Feb 2017).

[50] Raab G Technical working paper 6: education data available within the scottish longitudinal study. 2013 http://calls.ac.uk/wp-content/uploads/LSCS-WP-6.pdf2014.

[51] Wood R, Clark D, King A, et al Novel cross-sectoral linkage of routine health and education data at an all-Scotland level: a feasibility study. The Lancet 2013;382(suppl 3):S10 10.1016/S0140-6736(13)62435-6

[52] Mackay DF, Wood R, King A, et al Educational outcomes following breech delivery: a record-linkage study of 456947 children. Int J Epidemiol 2015;44:209–17. 10.1093/ije/dyu270 25613426 PMC4415090

[53] Tweed EJ, Mackay DF, Nelson SM, et al Five-minute Apgar score and educational outcomes: retrospective cohort study of 751,369 children. Arch Dis Child Fetal Neonatal Ed 2016;101:F121–F126. 10.1136/archdischild-2015-308483 26297221

[54] Gayle V, Berridge D, Davies R Young People's Entry into Higher Education: Quantifying influential factors. Oxf Rev Educ 2002;28:5–20. 10.1080/03054980120113607

[55] Scottish Government. High Level Summary of Statistics data for School Education trends In: High level summary of statistics trends - Chart data, ed., 2015, 2015 Ind Schools - Data.

[56] Scottish Council of Independent Schools. Pupil number comparisons by local authority area 2011/12. 2012 http://www.scis.org.uk/assets/Uploads/Facts-and-Statistics/2011-Local-Authority-comparisons-by-region.pdf (accessed Sep 2015).

[57] Scottish Government. Summary statistics for schools in Scotland, no.2 ¦ 2011 edition. Scotland: Scottish Government, 2012.

[58] Bhattacharya S, Lowit A, Bhattacharya S, et al Reproductive outcomes following induced abortion: a national register-based cohort study in Scotland. BMJ Open 2012;2:e000911 10.1136/bmjopen-2012-000911 PMC4400701 22869092

